# Supplementary material for: AI-Dentify: deep learning for proximal caries detection on bitewing x-ray - HUNT4 Oral Health Study
Source: BMC Oral Health. 2024 Mar 18;24:344. doi: 10.1186/s12903-024-04120-0 (PMC10946166; doi:10.1186/s12903-024-04120-0)
Supplement: Supplementary file 1 — Supplementary Material 1. [file 12903_2024_4120_MOESM1_ESM.pdf]

Additional materials 1: AI-Dentify: Deep learning for proximal caries detection on bitewing x-ray - HUNT4 Oral Health Study

Javier Pérez de Frutos*[1], Ragnhild Holden Helland[1], Shreya Desai[2], Line Cathrine Nymoen[4,5], Thomas Langø[1], Theodor Remman[3], and Abhijit Sen[4,5]

[1]Department of Health Research, SINTEF Digital, Professor Brochs gate 2, 7030, Trondheim, Norway
[2]Boneprox A.B., Gothenburg, Sweden
[3]Boneprox A.S., Tønsberg, Norway
[4]Department of public Health and Nursing, Norwegian University of Sciecne and Technology, Trondheim, Norway
[5]Kompetansesenteret Tannhelse Midt (TkMidt), Trondheim, Norway

March 5, 2024

1 Annotation procedure

Proximal caries were annotated by six different dental clinicians. Five of them associated with TkMidt research centre, NTNU, (Trondheim, Norway); and the sixth to Boneprox A.B., (Gothenburg, Sweden). The annotations consisted of labelled bounding boxes. The lesions were classified following Espelid and Tveit criteria [1], [2] into grades one to five. An additional label was used to identify caries whose grade cannot be clearly determined. Observers could also label an image as free of caries or reject it because of poor quality e.g., bad angulation, or wrong acquisition settings. As pointed out by the annotators, the biggest challenge was the diagnosis solely based on the X-ray image, as it was impossible to perform a visual inspection of the patient, to better identify the severity of the caries.

The AnnotationWeb [3] web based application was used to collect the observations. The annotation work was divided in three phases. Initially, each observer was given the task of annotating 200 selected bitewing images to assess the interobserver variability (see Section 1.1). Next, they were asked to annotate the complete dataset, where the images were randomly sampled from the dataset. Due to schedule restrictions, when the mark of 13,000 annotated images was reached, regardless of the number of observers that analysed the images, the dental clinicians were given a new task to only annotate those images out of the 13,000 that they have not viewed before. Additionally, the observers were brought together to collaboratively annotate a subset of images resulting in the consensus test set of 197 images.

1.1 Interobserver analysis

Prior the work done on the consensus test set, a one-to-one comparison was made instead to assess the variability between the annotators. The results are included for completeness. The goodness of agreement was measured using Cohen's Kappa (κ)[4], defined as

$$\kappa = 1 - \frac{1 - p_0}{1 - p_e} \tag{1}$$

where,

p_0 is the probability of agreement between the observers,

*Corresponding author (✉ javier.perezdefrutos@sintef.no, ☎ (+47) 982 45 167)

p_e is the probability of agreement by change between the annotators.

Notice how Cohen's Kappa ranges from -1 (worse than random agreement) to $+1$ (total agreement).

The bounding boxes drawn by the observers were then paired based on the intersection over union (IoU) score (see Equation (2)). Hence, κ was evaluated using different IoU thresholds.

$$IoU(A,B) = \frac{A \cap B}{A \cup B} \tag{2}$$

κ values for the six annotators are shown in Figure 1 for the IoU thresholds 0.2, 0.5 and 0.8, respectively.

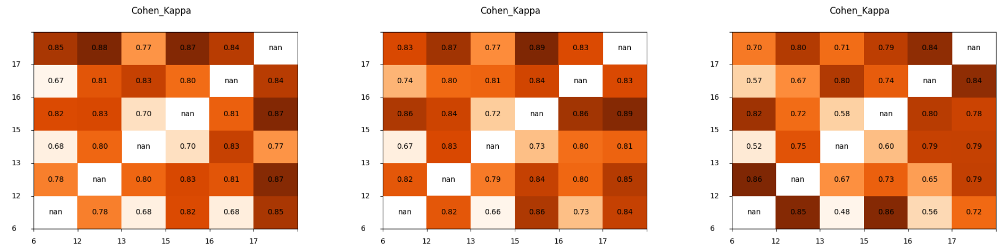

(a) (b) (c)

Figure 1: Cohen's Kappa values for different IoU thresholds: (a) 0.2, (b) 0.5, and (c) 0.8. Only grades one to five, and secondary lesions were considered.

Furthermore, the lesions were grouped in more general categories, as described in the manuscript: caries of grades one and two were classified as "enamel caries", whereas grades three to four were grouped as "dentine caries". Secondary caries and unknown caries were kept as their own category. The results same analysis on the agreement level of the annotators can be seen in Figure 2.

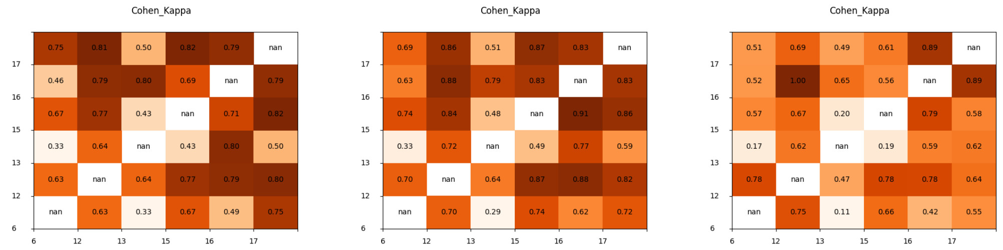

(a) (b) (c)

Figure 2: Cohen's Kappa values for different IoU thresholds: (a) 0.2, (b) 0.5, and (c) 0.8. Only enamel and dentine caries macro groups were considered.

2 Pipeline to process multi-observer object detection annotations

The strategy followed to combine the annotations comprised of two steps. First, the annotations in each image are assigned to different groups based on the IoU (see Algorithm 1). Next, the preassigned groups are analysed to build the "common bounding box" as described in Algorithm 2.

For each box in the group, a Gaussian distribution is fitted along the two axes of the image i.e., the vertical and horizontal axes. The probability density function (PDF) of these distributions are then combined, resulting in the mixture density function of a Gaussian Mixture Model where all distributions have the same weight. Given a confidence value (p), the mixture density is used to get that box which overlaps with the group as illustrated in Figure 3. The Gaussian distributions are fitted so the standard deviation is a fourth of the length of the box (see Figure 3).

3 Architectures

Details of the architectures used in this study are described in this section.

Algorithm 1 Group annotations

```
Inputs:
    L                                   ▷ List of annotations of an image
    k                                   ▷ IoU threshold
Initialize:
    A ← L{0}
    L ← L{1, ..., n}
    T[annotation, group]   ▷ Table where to save the group assignment for each
    annotation, group_counter ← 0
for i := 1 to n do
    B ← L{i}
    if IoU(A, B) ≥ k ∨ A ⊂ B ∨ B ⊂ A then
        if T(A) ≠ ∅ ∧ T(B) ≠ ∅ then
            if T(A).group ≠ T(B).group then      ▷ A and B are assigned to different groups
                Compare each annotation a in T(A) with each annotation b in T(B)
                if ∀(a, b), IoU(a, b) ≥ k then
                    T(∀a).group ← T(B).group   ▷ Annotations in the group of A are assigned to
the group of B
                else
                    Mark T(A) and T(B) to be manually inspected
                end if
            end if
        else if T(B).group ≠ ∅ then
            T(A).group ← T(B).group
        else if T(A).group ≠ ∅ then
            T(B).group ← T(A).group
        end if
    else
        T(A).group ← group_counter
        T(B).group ← group_counter
        group_counter ← group_counter + 1
    end if
end for
```

Algorithm 2 Combine bounding boxes

```
Inputs:
    B            ▷ List of bounding box coordinates (X, Y, W, H), with |B| boxes
    p                      ▷ p − value ∈ [0, 1] threshold for the GMM probability
lower_th = (1−p)/2
upper_th = (1+p)/2
for b in B do
    G_w ← NormalDistribution(μ = b.X + b.W/2, σ = b.W/4)
    G_h ← NormalDistribution(μ = b.Y + b.H/2, σ = b.H/4)
end for
for x in {w, h} do
    x_sampling       ▷ Evenly distributed sampling values, covering the combined domains of the
distributions in G_x
    for g in G_x do
        PDF_x ← ProbabilityDensityFunction(g, x_sampling)
    end for
    mixtureDensity_x[k] ← 1/|B| Σ_{i=0}^{|B|} PDF_x[i][k]
    cumulativeMD_x[k] = Σ_{i=0}^{k} mixtureDensity_x[i]      ▷ Cumulative sum of mixtureDensity_x
    x_min ← x_sampling(max(where(cumulativeMD_x ≤ lower_th)))
    x_max ← x_sampling(max(where(cumulativeMD_x ≤ upper_th)))
end for
```

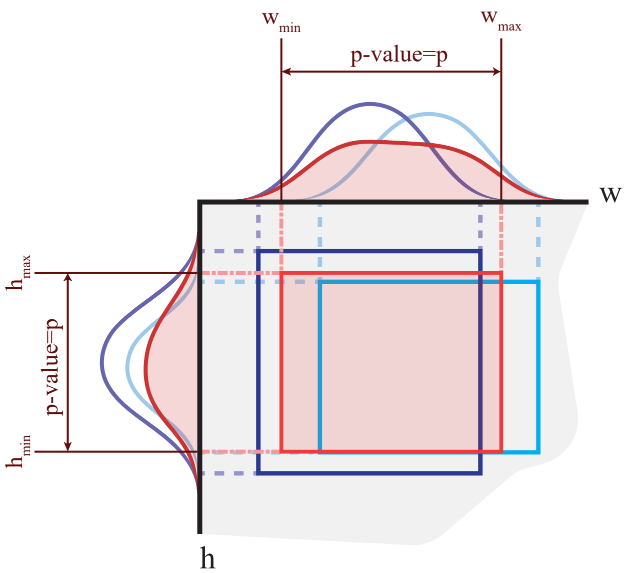

Figure 3: The common bounding box (red) is obtained from the combined PDF of the Gaussian distribution fitted to the two blue boxes.

3.1 RetinaNet

Firstly introduced in Lin, Goyal, Girshick, *et al.* [5], RetinaNet fashions a ResNet50 [6] backbone network connected with a feature pyramid network (FPN), providing multi-scale features. The backbone is usually initialised using the weights resulting of training on the ImageNet dataset [7]. As shown in Figure 4, two parallel fully connected networks are connected to each level of the FPN. Wheres the first sub-network is tasked to regress the anchor boxes, the second is entrusted with classifying these anchor boxes. The predictions are then combined using non-maximal suppression. The average precision reported on the COCO test-dev split showed better performance than Faster-R-CNN-two-stage detectors, and one-stage methods like YOLOv2 or SSD513 detectors. Moreover, the runtime reported was improved with respect to the baseline methods. Hence why it was included in this study.

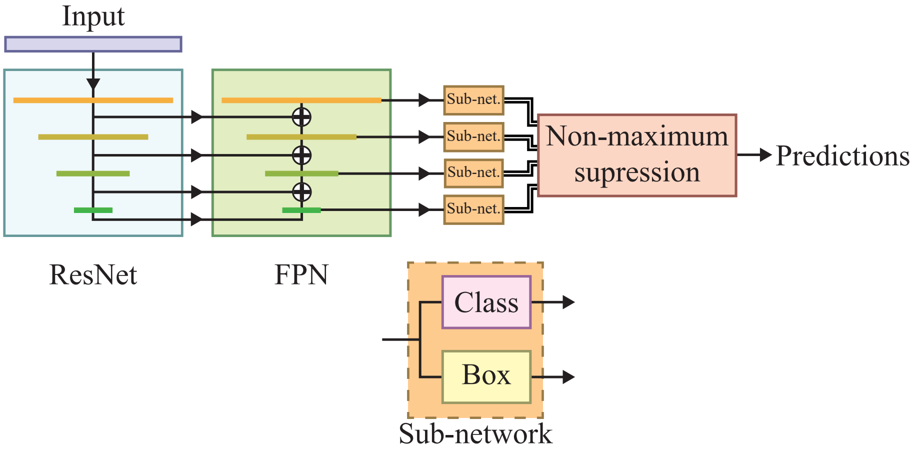

Figure 4: RetinaNet architecture, using ResNet as backbone, a feature pyramid network as neck, and two fully convolutional networks as head of the detector.

3.2 YOLOv5

YOLO architecture was first introduced in 2015 [8], since then, new updated version have been released, being v5 developed by Jocher, Chaurasia, Stoken, *et al.* [9], the one tested in this study. Like its former versions, is a one stage object detector with a similar architecture as GoogLeNet [10]. As in YOLOv4, YOLOv5 uses CSPDarknet53 [11] as backbone. This consists of an enhanced

fully-convolutional DarkNet53 [12] with the feature splitting strategy proposed in the cross stage partial DenseNet [13].

YOLOv5 architecture comes in five different sizes,increasing the number of parameters between each version: N (extra small), S (small), M (medium), L (large), X (extra large). Pretrained weights for each size are available to use in the original repository `https://github.com/ultralytics/yolov5`. The performance is shown to increase with the size of the network, expected with the increase in number of parameters, making it able to abstract more complex and relevant features. However, this improvement in performance come at expense of the runtime, increasing from 45 ms (YOLOv5n) up to 766 ms (YOLOv5x) [9]. In addition, a version with larger input size (identified with a 6) of 1280 pxwere made available. YOLOv5 sizes *S6* and *M6* were deemed an appropriate compromise between flexibility and resources for the presented application, and better suited to the image size of our dataset. Out of the five sizes, only the medium model was used in this study due to limitations in time and resources.

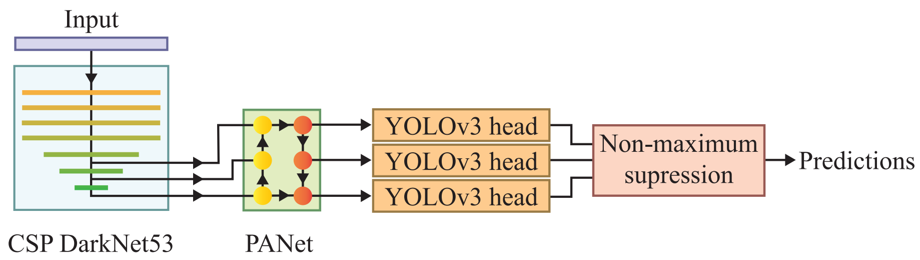

Figure 5: YOLOv5, using CSP DarkNet53 as backbone, followed by the Path Aggregator Network, and YOLOv3 head detector.

A schematic view of the YOLOv5 architecture is depicted in Figure 5. CSP Darknet-53 is used as the backbone model, featuring the cross stage partial (CSP) network strategy [13] which prevents redundant gradients, reducing memory requirements, and vanishing gradients by introducing skip connections through stages. The neck of YOLOv5 consists of a path aggregator network (PA Network), which combines the multi-scale features, enhanced with CSPNet blocks. Lastly, the same head block as in YOLOv3 is used to predict the coordinates of the bounding boxes, the label, and the prediction scores. Whereas the convolutions in the hidden layers of YOLOv5 feature the swish activation function i.e.g, sigmoid linear unit, the sigmoid function is used in the output layer instead.

3.3 EfficientDet

Developed by Tan, Pang, and Le [14], EfficientDet is a collection of object detection networks, built using EfficientNet [15] as backbone and pretrained on the ImageNet dataset [7]. The architecture features and encoder, with seven down sampling steps, connected to a bidirectional feature pyramid network (BiFPN), as shown in Figure 6. The output is then fed to a class and a box prediction networks, both sharing the same fully connected network architecture, which provide the object detection predictions. Non-maximal suppression is used to regress the final predictions.

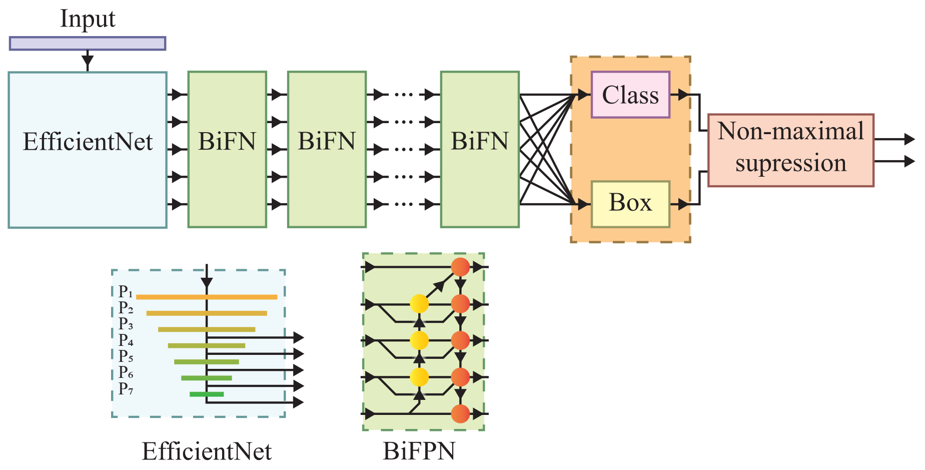

Figure 6: EfficientDet architecture, with EfficientNet as backbone, a bidirectional feature pyramid network (BiFPN) as neck, and two full connected networks as head of the detector.

The architecture is designed to scale up/down, following a compound factor (ϕ), as in EfficientNet [15]. A total of eight versions of pre-trained EfficientDet were made available: D0 to D7, each using the corresponding EfficientNet-B0 to B6 backbone. Due to time and resources restrictions, only the models for EfficientDet D0 and D1 were included in this study.

4 Extended results

Table 1 shows the per fold cross-validation (CV) evaluation results of the models, on the consensus dataset. Table 2 shows the evaluation results of both the trained models and the dental clinicians per class, aside from the average across classes. To ease the interpretation of these values, a graphical representation is shown in Figure 7.

Table 1: Cross-validation folds evaluation of the models on the consensus test set, with an IoU-threshold of 0.3. All metrics are reported as score over the whole test set.

Model	AP Enamel caries	AP Dentine caries	AP Secondary lesion	mAP	F1 Enamel caries	F1 Dentine caries	F1 Secondary lesion	mF1	FNR Enamel caries	FNR Dentine caries	FNR Secondary lesion	mFNR
YOLOv5, fold 1	0.635	0.645	0.714	0.665 ± 0.035	0.514	0.582	0.548	0.548 ± 0.028	0.114	0.200	0.128	0.147 ± 0.038
YOLOv5, fold 2	0.636	0.665	0.674	0.658 ± 0.016	0.514	0.622	0.556	0.564 ± 0.044	0.133	0.179	0.149	0.154 ± 0.019
YOLOv5, fold 3	0.555	0.604	0.660	0.606 ± 0.043	0.506	0.594	0.618	0.573 ± 0.048	0.200	0.221	0.191	0.204 ± 0.012
YOLOv5, fold 4	0.568	0.585	0.658	0.604 ± 0.039	0.532	0.570	0.533	0.545 ± 0.018	0.200	0.274	0.149	0.208 ± 0.051
YOLOv5, fold 5	0.588	0.609	0.701	0.633 ± 0.049	0.499	0.571	0.561	0.544 ± 0.032	0.152	0.200	0.149	0.167 ± 0.023
RetinaNet, fold 1	0.443	0.306	0.347	0.366 ± 0.057	0.187	0.315	0.206	0.236 ± 0.056	0.181	0.400	0.181	0.254 ± 0.103
RetinaNet, fold 2	0.407	0.317	0.380	0.368 ± 0.038	0.220	0.324	0.256	0.267 ± 0.043	0.162	0.421	0.245	0.276 ± 0.108
RetinaNet, fold 3	0.358	0.337	0.340	0.345 ± 0.009	0.262	0.282	0.279	0.275 ± 0.009	0.267	0.347	0.255	0.290 ± 0.041
RetinaNet, fold 4	0.452	0.331	0.391	0.391 ± 0.049	0.217	0.321	0.238	0.259 ± 0.045	0.152	0.358	0.149	0.220 ± 0.098
RetinaNet, fold 5	0.398	0.376	0.396	0.390 ± 0.010	0.256	0.318	0.192	0.255 ± 0.051	0.238	0.295	0.096	0.210 ± 0.084
EfficientDet D0, fold 1	0.266	0.282	0.261	0.270 ± 0.009	0.439	0.435	0.408	0.428 ± 0.014	0.552	0.663	0.681	0.632 ± 0.057
EfficientDet D0, fold 2	0.284	0.280	0.349	0.304 ± 0.032	0.448	0.446	0.543	0.479 ± 0.045	0.505	0.632	0.532	0.556 ± 0.055
EfficientDet D0, fold 3	0.284	0.270	0.276	0.277 ± 0.006	0.451	0.493	0.413	0.453 ± 0.033	0.514	0.611	0.660	0.595 ± 0.060
EfficientDet D0, fold 4	0.262	0.307	0.282	0.283 ± 0.018	0.445	0.471	0.447	0.454 ± 0.011	0.552	0.621	0.617	0.597 ± 0.031
EfficientDet D0, fold 5	0.239	0.254	0.384	0.292 ± 0.065	0.434	0.449	0.515	0.466 ± 0.035	0.543	0.653	0.543	0.579 ± 0.052
EfficientDet D1, fold 1	0.335	0.366	0.376	0.359 ± 0.018	0.507	0.553	0.543	0.534 ± 0.020	0.457	0.558	0.457	0.491 ± 0.047
EfficientDet D1, fold 2	0.314	0.374	0.370	0.352 ± 0.028	0.524	0.543	0.511	0.526 ± 0.013	0.438	0.537	0.489	0.488 ± 0.040
EfficientDet D1, fold 3	0.320	0.347	0.353	0.340 ± 0.014	0.471	0.540	0.528	0.513 ± 0.030	0.467	0.505	0.500	0.491 ± 0.017
EfficientDet D1, fold 4	0.336	0.418	0.357	0.370 ± 0.035	0.486	0.582	0.516	0.528 ± 0.040	0.495	0.516	0.489	0.500 ± 0.011
EfficientDet D1, fold 5	0.395	0.432	0.430	0.419 ± 0.017	0.549	0.588	0.570	0.569 ± 0.016	0.438	0.505	0.457	0.467 ± 0.028

Table 2: Evaluation of the models and individual annotators on the consensus test set, with an IoU-threshold of 0.3. All metrics are reported as score over the whole test set, and a 95% confidence interval.

Model / Annotator	AP Enamel caries	AP Dentine caries	AP Secondary lesion	mAP	F1 Enamel caries	F1 Dentine caries	F1 Secondary lesion	mF1	FNR Enamel caries	FNR Dentine caries	FNR Secondary lesion	mFNR
YOLOv5, fold 4	0.594 [0.483, 0.696]	0.622 [0.466, 0.733]	0.724 [0.622, 0.793]	0.647 [0.566, 0.707]	0.500 [0.435, 0.561]	0.576 [0.495, 0.655]	0.567 [0.500, 0.645]	0.548 [0.506, 0.598]	0.144 [0.082, 0.223]	0.183 [0.103, 0.291]	0.121 [0.071, 0.218]	0.149 [0.110, 0.203]
RetinaNet, fold 4	0.417 [0.317, 0.507]	0.404 [0.310, 0.486]	0.401 [0.299, 0.478]	0.407 [0.355, 0.458]	0.184 [0.151, 0.216]	0.229 [0.183, 0.272]	0.118 [0.096, 0.149]	0.177 [0.154, 0.202]	0.238 [0.162, 0.333]	0.295 [0.204, 0.396]	0.096 [0.046, 0.169]	0.210 [0.167, 0.262]
EfficientDet D0, fold 4	0.279 [0.173, 0.420]	0.321 [0.204, 0.418]	0.481 [0.344, 0.596]	0.360 [0.290, 0.431]	0.466 [0.372, 0.564]	0.520 [0.419, 0.616]	0.581 [0.469, 0.682]	0.522 [0.461, 0.588]	0.467 [0.351, 0.570]	0.560 [0.459, 0.664]	0.427 [0.324, 0.560]	0.484 [0.422, 0.552]
EfficientDet D1, fold 4	0.466 [0.309, 0.576]	0.482 [0.369, 0.591]	0.561 [0.399, 0.677]	0.503 [0.421, 0.569]	0.466 [0.309, 0.576]	0.482 [0.369, 0.591]	0.561 [0.399, 0.677]	0.503 [0.421, 0.569]	0.337 [0.241, 0.466]	0.447 [0.345, 0.558]	0.292 [0.191, 0.411]	0.359 [0.306, 0.431]
Annotator 1*	0.303 [0.240, 0.454]	0.258 [0.159, 0.348]	0.291 [0.210, 0.369]	0.284 [0.231, 0.347]	0.487 [0.397, 0.575]	0.495 [0.402, 0.588]	0.504 [0.440, 0.566]	0.495 [0.447, 0.552]	0.562 [0.449, 0.668]	0.495 [0.382, 0.602]	0.383 [0.274, 0.505]	0.480 [0.413, 0.552]
Annotator 2	0.243 [0.238, 0.302]	0.268 [0.225, 0.371]	0.239 [0.199, 0.331]	0.250 [0.247, 0.285]	0.357 [0.286, 0.416]	0.430 [0.367, 0.496]	0.369 [0.313, 0.425]	0.385 [0.346, 0.420]	0.400 [0.304, 0.506]	0.358 [0.246, 0.470]	0.170 [0.098, 0.267]	0.309 [0.251, 0.374]
Annotator 3	0.239 [0.181, 0.377]	0.136 [0.065, 0.251]	0.352 [0.261, 0.493]	0.242 [0.199, 0.320]	0.423 [0.341, 0.503]	0.250 [0.132, 0.390]	0.537 [0.441, 0.635]	0.403 [0.343, 0.470]	0.467 [0.351, 0.573]	0.853 [0.747, 0.927]	0.574 [0.451, 0.667]	0.631 [0.564, 0.686]
Annotator 4	0.212 [0.172, 0.311]	0.347 [0.293, 0.479]	0.339 [0.274, 0.427]	0.299 [0.270, 0.353]	0.360 [0.299, 0.431]	0.507 [0.440, 0.572]	0.483 [0.413, 0.544]	0.450 [0.411, 0.492]	0.352 [0.244, 0.452]	0.253 [0.164, 0.352]	0.106 [0.056, 0.189]	0.237 [0.180, 0.292]
Annotator 5	0.263 [0.202, 0.389]	0.226 [0.161, 0.315]	0.376 [0.301, 0.473]	0.288 [0.244, 0.356]	0.440 [0.350, 0.525]	0.442 [0.361, 0.517]	0.556 [0.491, 0.620]	0.479 [0.423, 0.528]	0.476 [0.353, 0.589]	0.600 [0.482, 0.694]	0.255 [0.166, 0.366]	0.444 [0.376, 0.515]
Annotator 6	0.255 [0.237, 0.306]	0.322 [0.297, 0.394]	0.204 [0.154, 0.255]	0.261 [0.248, 0.301]	0.345 [0.294, 0.398]	0.446 [0.394, 0.504]	0.338 [0.286, 0.383]	0.376 [0.346, 0.410]	0.143 [0.085, 0.214]	0.211 [0.127, 0.329]	0.138 [0.071, 0.233]	0.164 [0.124, 0.217]

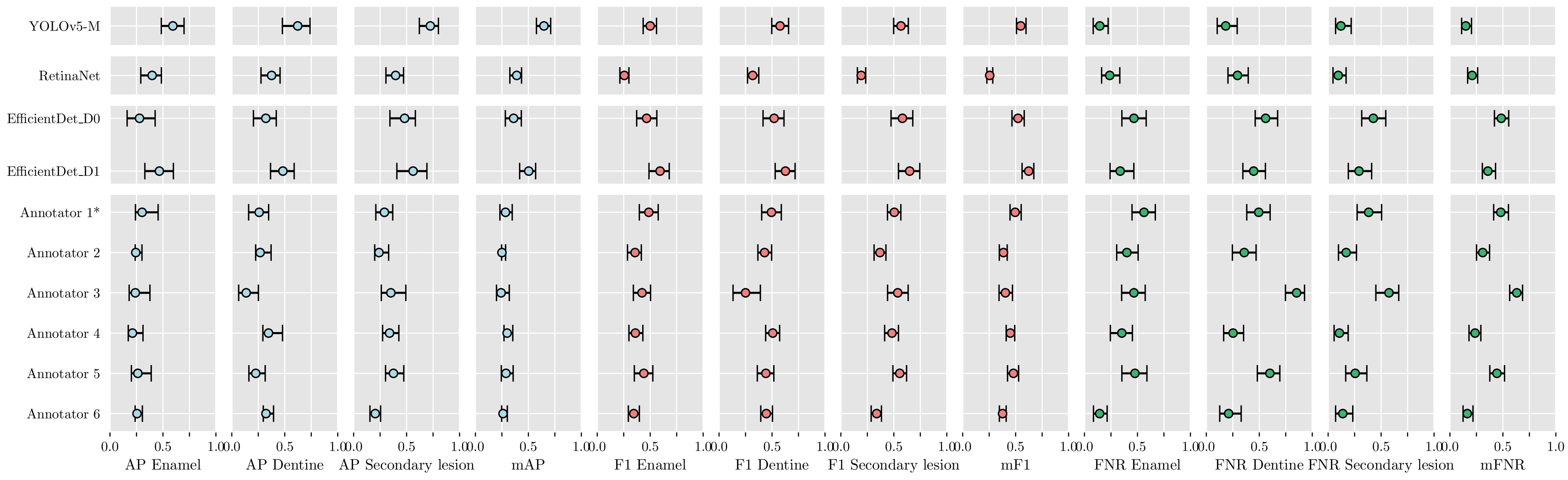

Figure 7: Bootstrap 95% confidence intervals for the metrics AP, F1 and FNR, for the models and the annotators
